# A Comparative Study of a 3D Bioprinted Gelatin-Based Lattice and Rectangular-Sheet Structures

**DOI:** 10.3390/gels4030073

**Published:** 2018-09-04

**Authors:** Shweta Anil Kumar, Nishat Tasnim, Erick Dominguez, Shane Allen, Laura J. Suggs, Yoshihiro Ito, Binata Joddar

**Affiliations:** 1 Inspired Materials & Stem-Cell Based Tissue Engineering Laboratory (IMSTEL), Department of Metallurgical, Materials and Biomedical Engineering, University of Texas at El Paso, 500 W University Avenue, El Paso, TX 79968, USA; sanilkumar@miners.utep.edu (S.A.K.); ntasnim@miners.utep.edu (N.T.); edominguez26@miners.utep.edu (E.D.); 2 Department of Biomedical Engineering, University of Texas at Austin, Austin, TX 78712, USA; shaneallen216@gmail.com (S.A.); suggs@utexas.edu (L.J.S.); 3 Nano Medical Engineering Laboratory, RIKEN Custer for Pioneering Research, RIKEN, 2-1 Hirosawa, Wako, Saitama 351-0198, Japan; y-ito@riken.jp; 4 Emergent Bioengineering Materials Research Team, RIKEN Center for Emergent Matter Science, 2-1 Hirosawa, Wako, Saitama 351-0198, Japan; 5 Border Biomedical Research Center, University of Texas at El Paso, 500 W University Avenue, El Paso, TX 79968, USA

**Keywords:** hydrogels, cardiac patches, 3D bioprinting, furfuryl-gelatin, lattice

## Abstract

3D bioprinting holds great promise in the field of regenerative medicine as it can create complex structures in a layer-by-layer manner using cell-laden bioinks, making it possible to imitate native tissues. Current bioinks lack both high printability and biocompatibility required in this respect. Hence, the development of bioinks that exhibit both properties is needed. In our previous study, a furfuryl-gelatin-based bioink, crosslinkable by visible light, was used for creating mouse mesenchymal stem cell-laden structures with a high fidelity. In this study, lattice mesh geometries were printed in a comparative study to test against the properties of a traditional rectangular-sheet. After 3D printing and crosslinking, both structures were analysed for swelling and rheological properties, and their porosity was estimated using scanning electron microscopy. The results showed that the lattice structure was relatively more porous with enhanced rheological properties and exhibited a lower degradation rate compared to the rectangular-sheet. Further, the lattice allowed cells to proliferate to a greater extent compared to the rectangular-sheet, which initially retained a lower number of cells. All of these results collectively affirmed that the lattice poses as a superior scaffold design for tissue engineering applications.

## 1. Introduction

The creation of cell patterns within specific spaces with the retention of their cell function and vitality, through 3D bioprinting, is a method that can be dated back to the 1990s [1]. This technique, with its vast potential and ceaseless possibilities, can bring about path-breaking changes in the field of regenerative medicine and therapeutics by generating complex tissues and organs that can be implanted in-vivo [1]. 3D bioprinting employs a “bioprinter” which uses a “bioink” that can exhibit the characteristics of an extracellular matrix environment and facilitate cell adhesion, proliferation, and differentiation [2,3,4]. Bioinks usually have the cells suspended in a hydrogel-like mixture and loaded within extrusion devices such as syringes prior to printing [5]. After printing, the structural fidelity of the printed shape is retained by secondary crosslinking mechanisms [6]. Traditional bioinks used for bioprinting do not generally possess high fidelity and biocompatibility, which fails to reproduce the complexity in biological organs or tissues [3,7]. So, in a recent study, we optimized the properties of a visible light crosslinkable gelatin-based bioink and successfully printed bilayer rectangular-sheet structures infused with C2C12 myoblasts and STO fibroblasts [6]. The furfuryl-gelatin (f-gelatin) crosslinks through visible-light irradiation, where O_2_ changes to ^1^O_2_ (singlet-oxygen) when the Rose Bengal dye acquires energy from visible-light [6]. The formation of singlet-oxygen leads to the formation of a stable cross-linked f-gelatin structure after bioprinting and deposition [6]. These bioprinted cell-sheets exhibited high fidelity during sustained in vitro culture and the encapsulated cells retained viability and exhibited heterocellular coupling [6]. However, the study did not provide any scope to assess the ability of this gelatin-based bioink to create complex structures such as a lattice, which may serve as an enhanced physical scaffold and provide microarchitectural cues necessary for mimicking the native architecture of the myocardium [8]. This subsequent study explored the feasibility of 3D bioprinting a lattice structure using the same f-gelatin-based bioink and compared it with the rectangular-sheet structure from the prior study by careful considerations of factors like structural fidelity, rheological properties, porosity, and cytocompatibility. We hypothesised that the experiments performed as a part of this study would help us to observe considerable differences between the two structures, i.e., lattice and rectangle, and also open up the possibility of significantly enhancing the design of a 3D bioprinted construct for engineering cardiac tissue-on-a-chip, using bioprinting.

## 2. Results and Discussion

**Figure 1:**
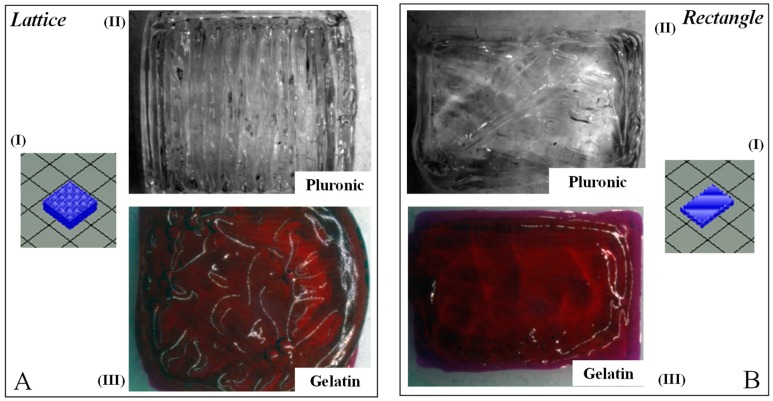
Gross Morphology of lattice and rectangular-sheet structures printed using pluronic and gelatin. (**AI**,**BI**) depict the stl. file image for the lattice and rectangular structures, respectively. (**AII**,**BII**) represent the en-face images for the same structures printed using Pluronic F-127. (**AIII**,**BIII**) are representations of the en-face images for lattice and rectangular patterns printed using the f-gelatin-based bioink, respectively.

A comparison of morphology between the two rectangular-sheet structures made using either pluronic or f-gelatin-based bioink revealed the following results. These two rectangular-sheet structures appeared similar in morphology and dimensions (Figure 1(BII,III)). On the other hand, the lattice structures fabricated using either pluronic or f-gelatin-based bioink revealed significant differences (Figure 1(AII,III)). The stereolithography (stl.) designs used for printing are depicted in Figure 1(AI,BI), respectively. The pluronic lattice structure retained the structural complexity and fidelity of the lattice greatly, compared with the f-gelatin-based structure (Figure 1(AII,III)).

**Figure 2:**
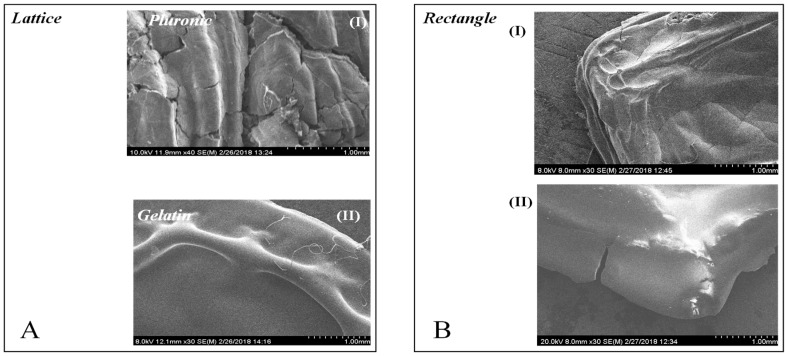
Representative SEM surface images of lattice and rectangular-sheet structures deposited using pluronic and gelatin. (**AI**,**II**) show the en-face images for the lattice structures printed with Pluronic-F127 and the f-gelatin-based bioink, respectively. (**BI**,**II**) depict the en-face images for the rectangular structures printed using the same materials, respectively.

Similarly, scanning electron microscopy (SEM) en-face images of both the lattice and rectangular-sheet revealed significant differences in lattice structures only, cast using pluronic and f-gelatin-based bioinks (Figure 2(AI,II). On the other hand, the rectangular-sheet structures made using either pluronic (Figure 2(BI)) or f-gelatin (Figure 2(BII)) exhibited structural resemblance. Low magnification images of lattice and rectangular structures made using f-gelatin are depicted in Supplementary Figure S1, to delineate the differences in both structures cast using the same bioink.

**Figure 3:**
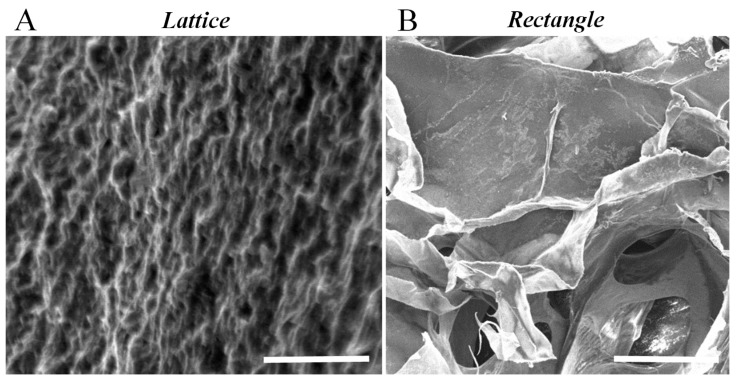
A representative SEM cross-section image of a gelatin lattice structure that was acquired in order to determine the apparent porosity and average pore size (**A**). The cross-sectional SEM image for the rectangular-sheet structure printed using f-gelatin was previously reported (**B**) [7]. The scale bars represent 5 µm and 500 µm in (**A**) and (**B**) respectively.

The SEM cross-sectional image of the gelatin lattice revealed a highly organized, striated, patterned, and networked structure (Figure 3A) in comparison to the loosely networked and largely porous rectangular-sheet cross-section SEM, as reported in our previous study (Figure 3B) [6]. Porosity and pore-size are crucial to ensure cell colonization of the scaffold, deposited using bioprinting. Likewise, SEM micrographs showed a homogeneous distribution of equal sized pores within the entire area scanned and imaged (Figure 3A).

Furthermore, the cross-section SEM image also showed well-interconnected pores with a mean value of 1 μm (Figure 3). This was significantly smaller compared to the average pore size of the rectangular-sheet structures, as reported in our previous study [6]. The average apparent porosity of this lattice structure was estimated to be about 50% compared to 21% for the rectangular-sheet [6]. The results led us to conclude that although the mean pore size was significantly reduced by printing in the form of a lattice, the inherent design of the lattice allowed pores to be of a similar size and to be homogenously distributed throughout the entire structure, compared with the rectangular-sheet [6].

**Figure 4:**
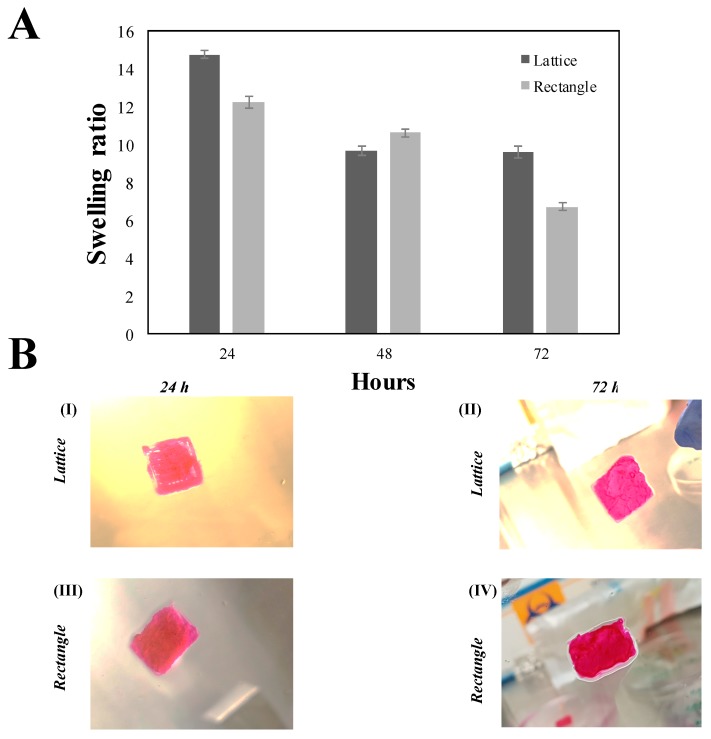
(**A**) Swelling analysis for both the f-gelatin-based lattice and rectangular-sheets over a period of three days after being subjected to visible light crosslinking. (**B**) Shown above in (**BI**,**II**) is cell-laden lattice constructs. In (**BIII,IV**) is cell-laden rectangular-sheet constructs. (**BI**,**III**) was acquired after 24 h of culture. (**BII**,**IV**) was acquired after 72 h of culture. No significant differences in the degradation rates of both structures were evident at 72 h of incubation.

The swelling kinetics and behavior of the bioprinted lattice and rectangular crosslinked hydrogels are depicted in Figure 4. For all samples tested, the maximum swelling was observed at 24 h, as reported in our previous study [6]. After 24 h of incubation, the structures seem to degrade slightly, as evidenced by 48 h (Figure 4A). However, the lattice structure seemed to have reached an equilibrium point after 48 h as it did not exhibit any further degradation beyond 48 h, when analyzed at 72 h (Figure 4A,B). On the other hand, the rectangular-sheet continued to degrade beyond 48 h, when analyzed at 72 h (Figure 4A,B). These observations confirmed that the lattice structures exhibited a lower degree of swelling compared to the rectangular-sheets and these results enabled us to conclude that the lattice posed a more stable structure compared with the latter (Figure 4A,B). No significant differences in trends between both structures were noted. Although data reported was from three days of observation and analysis, the stored gels did not degrade until six days in culture following this observation.

**Figure 5:**
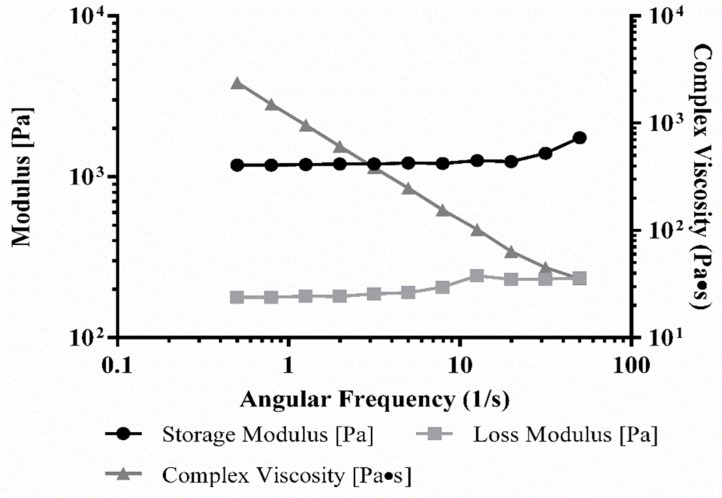
Rheology analysis of f-gelatin-based lattice structures, obtained from a disc-shaped (8 mm) sample.

From the rheometric analysis, it was established that the strain and frequency range were in the linear viscoelastic range of the gels by amplitude and frequency sweeps (Figure 5). The crosslinked gels exhibited an elastic modulus of 5.5 ± 2.4 kPa and complex viscosity of 920 ± 400 Pa.s, both of which were significantly higher, ~5 times, compared to the respective values of the rectangular sheet structure [6]. These results implied that although the bioink material composition and the crosslinking mechanism were unaltered, the lattice geometry actually enhanced the stiffness and mechanical properties of the printed structures. However, the larger error is due to the fact that the lattice structures, being macroporous, were more difficult to measure with shear.

**Figure 6:**
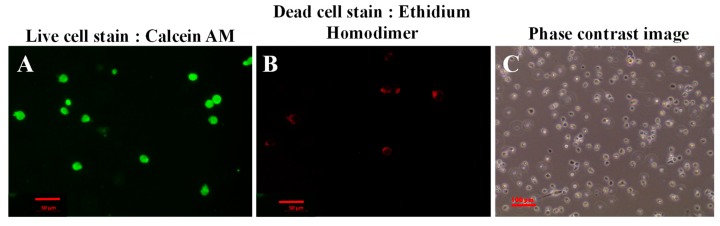
Live/Dead assay performed 15 min after printing and crosslinking. Shown in (**A**) are calcein stained live cells and in (**B**) are ethidium homodimer stained dead cells, respectively. In (**C**), a phase-contrast image of cells cultured for this experiment is shown.

Live/Dead assay results showed evidence of fewer dead cells compared to a greater number of live cells, after bioprinting and crosslinking of the printed structure (Figure 6). The number of live cells was significantly more (*p* = 0.03) at 22 ± 5 per unit area (12 × 10^4^ sq. microns) compared to the number of estimated dead cells per unit area (6 ± 2). Thus, the average percentage cell-viability was estimated to be 81.48% compared to a low percentage of cell death, averaging only 22%. This data was in the range of the results shown by others during cell bioprinting via extrusion methods [9]. 

**Figure 7:**
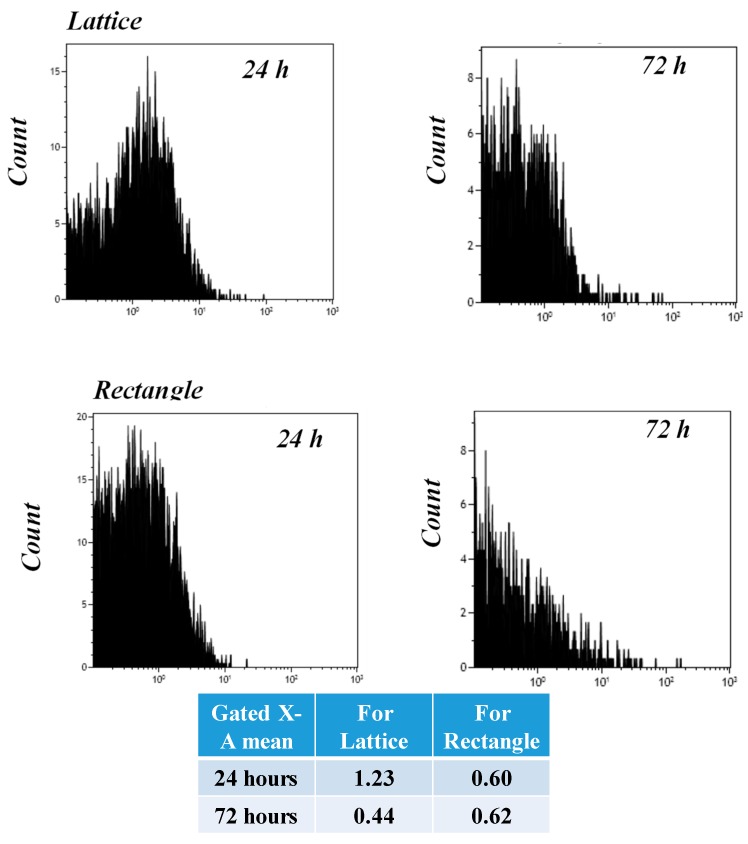
FACS analysis to show cell proliferation and biocompatibility of the printed and crosslinked lattice and rectangular-sheet structures, respectively. Cells pre-stained with CTV were cultured up to 24 and 72-h within printed constructs, respectively. Gated X-A mean values indicate the average intensity of the dye exhibited during that particular sample run.

To estimate cell proliferation in the bioprinted lattice and rectangular-sheet structures, the cells were pre-stained using Cell Trace Violet (CTV), a proliferation kit (Invitrogen, Carlsbad, CA, USA), and subjected to Flow Cytometry (FACS) analysis. The principle of use governing the application of the dye, CTV, to track cell proliferation is based on an underlying concept of dye dilution which allows several generations of cells to be analyzed using just one-time staining of the cells, prior to culture [10]. As the cells proliferate, the dye intensity gets diluted with increasing generations of cells produced within the same culture. Gated X-A mean usually refers to the intensity of the dye used for cell tracking and proliferation [10]. So, a higher value of the Gated X-A mean indicates less dye dilution and generations of cells, and a lower value indicates enhanced dye dilution and an increase in the generation of cells, respectively. Thus, the use of dye dilution assays on asynchronously growing cell lines is a potentially powerful method for tracking cell proliferation [11]. Results from FACS analysis showed that from 24 till 72-h of culture, cells cultured within the lattice showed enhanced proliferation compared to the rectangular-sheet (Figure 7). This is because the Gated X-A mean was significantly reduced in its value from 24 till 72-h in cells cultured within the lattice (1.23 versus 0.44, respectively) compared to the rectangle, which did not reveal significant differences from 24 till 72-h (0.60 versus 0.62, respectively). Further, the occurrence of multiple peaks (Figure 7) confirmed the presence of consecutive proliferating generations of cells in the bioprinted constructs. The positive controls consisting of CTV stained cells cultured on plastic for 24 and 72-h also showed enhanced dye dilution and cell generations from 24 till 72-h, as indicated by their Gated X-A mean values (Supplementary Figure S2). In summary, the lattice structures allowed cells to connect and communicate better, resulting in higher cell growth indicated by the greater extent of dye dilution, compared to the rectangular-sheet structures. These results were cross verified from absolute cell counts, as described. For the 3D printed constructs, 2 × 10^5^ cells/mL were used for cell encapsulation. Since the lattice had a larger volume (1.5 cm × 1.5 cm × 1 mm) than the rectangular sheet (1.5 cm × 1 cm × 1 mm), more cells could be entrapped within the lattice compared to the rectangle when analyzed after 24 h, at which time the lattice revealed a cell density of 4.2 × 10^5^ cells/mL compared to the rectangular sheet which had a value of 2.4 × 10^5^ cells/mL. After 72 h, cells in both samples had proliferated about four times compared to the initial cell seeding density. In our previously published study [6], the CTV dye was used at a 1:1000 dilution and so the proportional decrease in the intensity of the dye with enhancing cell populations could not be well detected. So, in this study, we chose to apply a 1:5000 dilution to detect the proportional decrease in the intensity of the dye, expressed by generations of proliferating cells.

**Figure 8:**
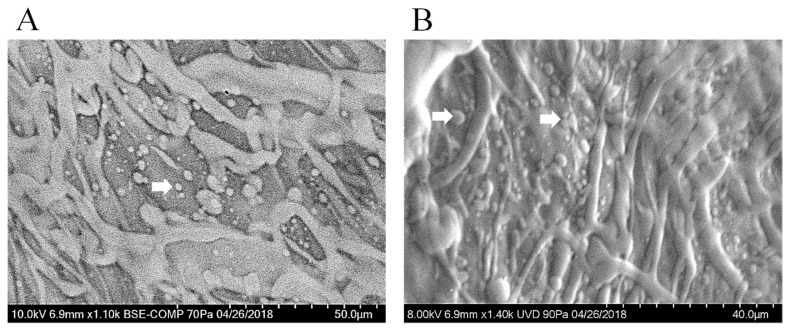
Shown in (**A**,**B**) are characteristic images of mouse MSC printed in lattice structures. Elongated cell morphologies and the extensive coverage area by the cells both confirm the biocompatibility of the lattice design and the bioink used. White arrows point to the extracellular matrix deposited by the cells cultured.

SEM images of cells in lattice structures revealed confluent cell populated surfaces, as shown in Figure 8A,B. Besides, there was also a significant amount of extracellular matrix (pointed using white block arrows) deposited by cells, noted in all representative images (Figure 8A,B), as is also supported by our previously published work [12]. These results implied that the lattice is a favorable scaffold design permissive towards cell growth and proliferation, owing to its macro-porosity (Supplementary Figure S3).

In summary, the lattice structure appeared to present itself as a more stable scaffold with enhanced structural rigidity, optimal porosity, and well-connected pores, which allowed the encapsulated cells to proliferate more, compared to the rectangular-sheet structure. This study revealed fundamental differences in two dissimilar, chemically cross-linked soft structures fabricated using the same bioink. This fundamental difference gave rise to divergent mechanical properties of both structures, which may have also influenced cell behavior, growth, and proliferation, enabling the lattice structure to allow cells to communicate better, eventually resulting in better cell yields compared to the rectangular-sheet. In addition, the lattice allows more bioink volume to be deposited, thereby allowing a greater number of cells to be encapsulated within the printed structure. Thus, the lattice structure poses as a better choice for a scaffold for creating 3D bioprinted structures for tissue engineering applications. The lattice is a multi-layered structure and in the future, it can be explored as a suitable platform for culturing multiple cell types within the same structure in a layer-by-layer fashion [13,14].

## 3. Materials and Methods

### 3.1. Chemicals

Furfuryl gelatin (f-gelatin), used as a basis for the “bioink”, was synthesized and characterized as described [15,16]. Hyaluronic Acid Sodium Salt (HA; mol. wt. ~1.5–1.8 × 10^6^ Da) was obtained from Sigma-Aldrich (St. Louis, MO, USA). Rose Bengal (RB), a visible light crosslinker, was procured from ThermoFisher Scientific (Waltham, MA, USA). Pluronic F127 was obtained as a “bioink” from Allevi (formerly Biobots, Philadelphia, PA, USA). 1X PBS was obtained as a sterile solution (ThermoFisher, Waltham, MA, USA). The Live/Dead Cytotoxicity Assay Kit Green/Red Staining kit was obtained and used following the manufacturer’s recommendations (Marker Gene Technologies Inc., Eugene, OR, USA). Dulbecco Modified Eagle’s Medium (DMEM) and 0.25% trypsin-EDTA were obtained from ThermoFisher. The Cell Trace Violet (CTV) proliferation kit was obtained from Invitrogen (Carlsbad, CA, USA). Cells and growth medium used in this study are enlisted in the subsequent section.

### 3.2. Cells and Growth Medium

Strain C57BL/6 Mouse Mesenchymal Stem Cells (mouse MSC, catalogue #: MUBMX-01001) were used to disperse in the “bioink” and printed into structures. For their culture, growth, and maintenance, Mouse Mesenchymal Stem Cell Growth Medium (complete growth medium, catalogue #: MUXMX-90011) was obtained from Cyagen, Santa Clara, CA, USA. Cells were cultured and passaged according to the manufacturer’s recommendations.

### 3.3. Biofabrication

For biofabrication, an ALLEVI 2 (formerly the BIOBOT 1, Allevi, Philadelphia, PA, USA), we formulated the “bioink” and cell mixture as optimized in our previous study, as described [6]. The bioink was made up of a mixture of f-gelatin, HA, and RB in quantities mentioned earlier [6,17,18]. Briefly, to make 1 mL of bioink, 10 mg of HA and 100 mg of f-gelatin was dissolved in DI water (900 µL, 25 °C) and mixed thoroughly by heating in a water bath (37 °C, 1 h) to render the formation of a homogenous viscous mixture. To this, 100 µL of RB (5% *w*/*v*) was added for crosslinking after bioprinting. Finally, cells were added to this gel-like mixture, loaded within a 10 mL plastic syringe (BD, Franklin Lakes, NJ, USA), fitted with a stainless steel blunt-tip dispensing needle (20 G, 0.6 mm diameter, Huaha, AZ, USA), and extruded using a low extrusion pressure in the range of 2.6–4.8 psi [6]. Two dissimilar patterns, namely a lattice and a rectangular-sheet [6], were used for printing structures, in 100 mm × 15 mm petri-dishes (ThermoFisher, Waltham, MA, USA). The dimensions of the lattice were 1.5 cm × 1.5 cm and the rectangular-sheet, 1.5 cm × 1 cm. Both structures were printed up to a thickness of 1 mm. After printing, the structures were exposed to visible light for 2.5 min to facilitate chemical crosslinking (400 nm wavelength at 100% intensity, Intelli-Ray 600, Uvitron International, West Springfield, MA, USA).

As a positive control for structural comparison, pluronic was used to mimic complex structures via bioprinting [3]. For printing, pluronic was maintained at room temperature (25 °C) until it freely flowed and then loaded within a 10 mL plastic syringe (BD, Franklin Lakes, NJ, USA), fitted with a stainless steel blunt-tip dispensing needle (23 G, 0.34 mm diameter) and extruded using a high extrusion pressure in the range of 25.9–29 psi, following recommendations from Allevi.

### 3.4. Gross Morphology

To compare and contrast the overall morphology and identify essential dissimilarities in both printed lattice and rectangular-sheet structures, digital images of structures printed using f-gelatin and pluronic were acquired using an upright Leica M205C microscope (Leica Microsystems, Buffalo Grove, IL, USA).

### 3.5. Rheology of Bioink

Rheological characterization was evaluated using a previous standardized approach for the proper analysis of hydrogel rheological properties. For measurement, printed and crosslinked gels (without cells) were pre-swollen in 1X PBS (1 h, 25 °C) before testing and cut out from the lattice and rectangular sheet structures, using a biopsy punch (~1 mm deep, 8 mm diameter). We performed oscillatory shear stress rheometry on these gel samples at 1% strain using a frequency range of 0.5–50 Hz on an Anton-Paar MCR101 rheometer (Anton-Paar, Graz, Austria) through an 8 mm parallel plate geometry. The strain and frequency range were examined in the linear viscoelastic range of the gels by frequency sweeps. Elastic modulus was calculated by a previously established formula using complex shear modulus with storage and loss modulus, corresponding to the complex viscosity measured at 1.99 Hz for all samples [6,19].

### 3.6. In-Vitro Culture Conditions for the Cell-Laden Constructs

The cell-laden, bioprinted, and crosslinked constructs of both lattice and rectangular-sheets were overlaid with complete growth medium for mouse MSC and cultured in an incubator (37 °C, 5% CO_2_ and 95% RH) up to 72 h. During this time, the culture medium was changed by removing the spent medium and by adding fresh growth medium after every 24-h interval. From our previous study, it was shown that culturing the cell-laden constructs in this manner did not affect cell viability nor proliferation, even in the interior parts of the construct, as it was reasonably porous, allowing nutrient uptake and oxygenation [6]. Moreover, 3D cell printing has been shown to allow the effective generation of a cell-laden porous architecture that enables a sufficient supply of cellular nutrition and oxygen [20]. In addition, others have shown that porous 3D cell-printed patches exhibit a higher cell viability [20] than their non-3D printed counterparts.

### 3.7. Live/Dead Cytotoxicity Assay

The cytotoxicity assay was performed using a Live/Dead assay kit, to assess the biocompatibility of the f-gelatin bioink and the printing method [21]. Mouse MSC after being subjected to extrusion-based bioprinting and visible light crosslinking were subjected to the Live/Dead assay (after 15 min). First, the cell-laden structures were rinsed with 1X PBS (three times) and supplemented with pre-warmed complete growth medium for an additional 15 min. Then, the growth medium was removed, and the samples incubated with pre-warmed cytotoxicity reagent (Live/Dead Cytotoxicity Assay Kit Green/Red Staining). A sufficient amount of the reagent was added to cover the samples during the incubation process, for which the samples were placed back into the incubator for 45 min. After incubation, the samples were washed using 1X PBS and imaged using light and confocal microscopy (ZEISS LSM 700 Confocal, Oberkochen, Germany). Acquired images were analyzed using Image J (NIH, Bethesda, MD, USA) and the number of live or dead cells per unit area was reported as the mean ± stdev.

### 3.8. Scanning Electron Microscopy

The porous texture of the lattice was analyzed by scanning electron microscopy (SEM) to ensure that the hydrogel retained its structure after printing and crosslinking. Cross-sectional and en-face images of the dried-gels prepared using the f-gelatin-based bioink were acquired using SEM, following published procedures [6,12]. For SEM, acellular and cell-laden samples were employed to determine the morphology and porous structure of the hydrogels, and cell behavior on the scaffolds, respectively.

For sample preparation, lattice structures were printed, dried in a desiccator (Nalgene, Sigma, St. Louis, MO, USA) overnight, and visualized using SEM. Prior to SEM imaging, samples were sputter-coated with gold (1 min, Au coating thickness of 5 nm) in a sputter coater (Jeol USA Smart Sputter Coater, JEOL USA, Inc., Peabody, MA, USA).

To calculate the average pore size and porosity of the lattice and rectangular-sheet structures, cross-sections were prepared for SEM imaging. As this step required a substantial amount of bioink material, only thick sections for the lattice structure were prepared as the data for the rectangular-sheet structures already exists [6]. For sample preparation, for imaging of the cross-section of a lattice structure printed using the f-gelatin-based bioink, the prepared samples were dried in a desiccator, sputter-coated, and visualized using SEM (S-4800, Hitachi, Tokyo, Japan). Cross-sectional SEM images obtained were analyzed using Image J to determine the average pore size and the apparent porosity (%), respectively. A detailed description is provided in the online supplementary information. Apparent porosity was calculated by the following Formulae (1):

(1) $$\;App.\;porosity=\frac{total\;area\;covered\;by\;pores\;\left(\mathrm{sq}.\mu m\;\right)}{total\;sample\;area\;of\;the\;cross-section\left(\mathrm{sq}.\mu m\right)}\;\times \;100$$

SEM was also done to confirm cell retention, spreading, and maintenance of viable cell morphology within the scaffolds, in addition to revealing the overall structure of the lattice scaffolds. For en-face imaging of samples with cells, the cross-section of one representative specimen from each sample pool was examined using a Hitachi TM-1000 SEM equipped with a backscattered electron detector and operating at an accelerating voltage of 15 kV (Hitachi High-Technologies Europe GmbH, Krefeld, Germany) [22].

### 3.9. Swelling Behaviour

The swelling behavior of the gels was monitored in Dulbecco Modified Eagle’s Medium (DMEM, pH = 7, 25 °C) for five days to study the hydration dynamics of the crosslinked hydrogel structure [6,12]. Two representative structures of one lattice and one rectangular-sheet each, were printed, crosslinked, and stored at −80 °C (12 h), following which the samples were dried overnight in a desiccator. These dried samples were weighed (*W*0) and then immersed in DMEM and the increased weight due to swelling and water intake was recorded periodically (*Wt*), after every 24 till 72-h. The swelling ratio was calculated using the following Equation (2), where *Ds* represents the degree of swelling, and *W*0 and *Wt* represent the weights of the samples in the dry and swollen states, respectively [6,12].

(2) $$\;Ds=\frac{Wt-W0\;}{W0}$$

### 3.10. Cell Proliferation

For an estimation of the actual cell numbers in a sample at different time points, lattice structures printed with cells and cultured as described earlier were carefully rinsed with PBS, overlaid with a generous amount of 0.25% trypsin-EDTA, and incubated at 37 °C for 10 min in an orbital shaker (45 rpm). Cells extracted from the first-trypsinization cycle were pelleted by centrifugation, added with other cells that were removed in a second cycle of trypsinization, and counted using a haemocytometer throughout the entire culture period after 24 and 48-h of culture. Absolute cell densities extracted from samples at 24 and 48-h are reported.

### 3.11. Flow Cytometry (FACS) Analysis

To estimate cell proliferation in the bioprinted lattice and rectangular-sheet structures, the cells were pre-stained using the Cell Trace Violet (CTV) proliferation kit using the manufacturer’s protocols [6,12]. Briefly, 1:5000 dilutions were used for the CTV dye in this study, for pre-staining cells. These pre-stained cells were mixed with the bioink (2 × 10^5^ cells/mL) and printed into a lattice or rectangular-sheets and cultured for 24 and 72-h, respectively (37 °C, 5% CO_2_). After 24 and 72-h, cell-gel samples were treated using Trypsin-EDTA (0.25%, phenol red), after which the cells were detached, extracted, and processed for flow cytometry (FACS). Extracted cells were fixed and processed further for FACS (Beckman Coulter Gallios Flow Cytometer, Brea, CA, USA) using excitation and emission wavelengths of 405 and 450 nm, respectively. Positive controls included pre-stained cells (1 × 10^5^ cells/mL) grown on plastic petri dishes for 24 and 72-h, respectively. Negative controls included non-stained cells grown on plastic petri dishes for 24 and 72-h, respectively.

## Supplementary Materials

The following are available online at http://www.mdpi.com/2310-2861/4/3/73/s1, Figure S1: Low magnification images of lattice and rectangular-sheets, Figure S2: FACS analysis of controls, Figure S3: Fabrication of a lattice structure. Supplementary methods are also included.
