# Supplementary material for: A Comparative Study of a 3D Bioprinted Gelatin-Based Lattice and Rectangular-Sheet Structures
Source: Gels. 2018 Sep 4;4(3):73. doi: 10.3390/gels4030073 (PMC6209247; doi:10.3390/gels4030073)
Supplement: Supplementary file 1 [file gels-04-00073-s001.pdf]

Supplementary Information

A Comparative Study of a 3D Bioprinted Gelatin-Based Lattice and Rectangular-Sheet Structures

Shweta Anil Kumar, Nishat Tasnim, Erick Dominguez, Shane C. Allen, Laura Suggs, Yoshihiro Ito, Binata Joddar

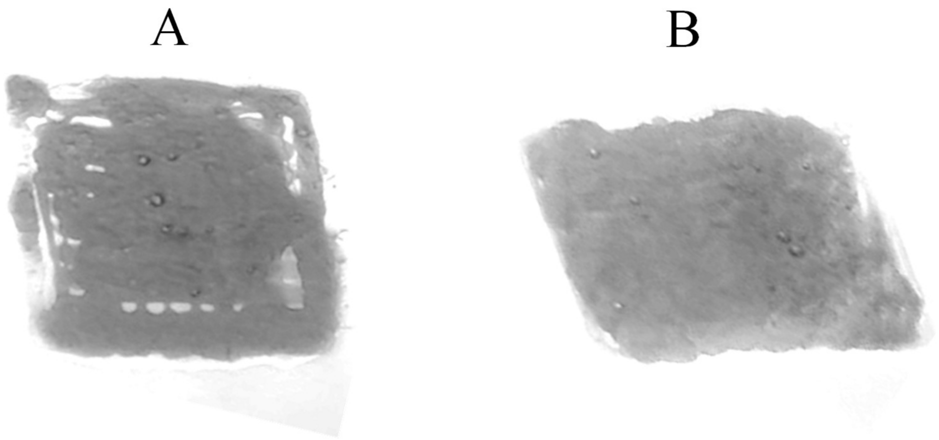

Supplementary Figure 1. Representative digital camera (low magnification images) B/W images of lattice (A) and (B) rectangular-sheet structures deposited using gelatin.

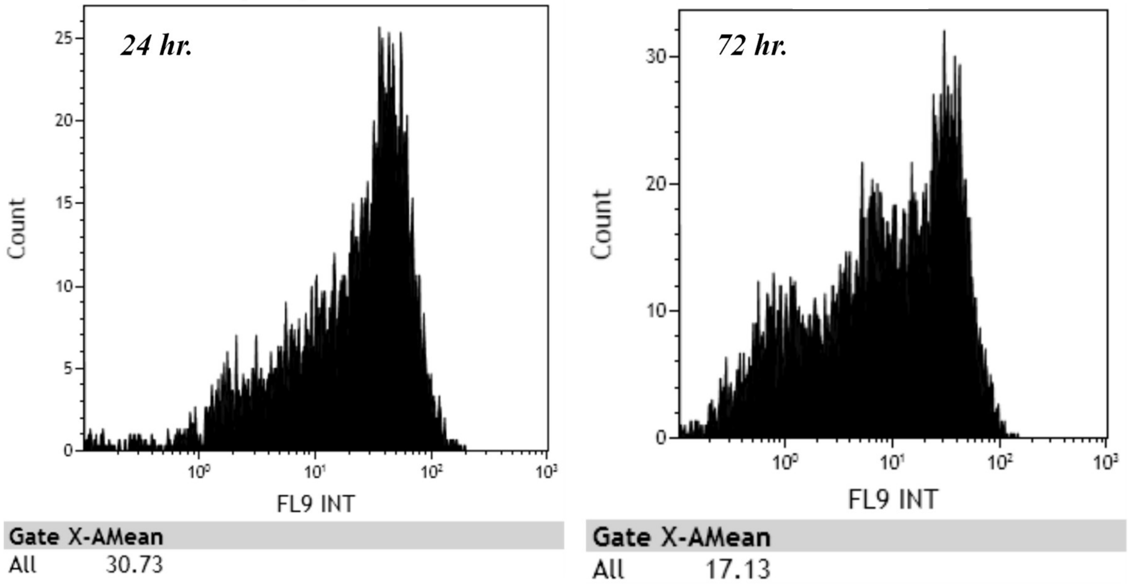

Supplementary Figure 2. FACS analysis of CTV pre-stained control cells not bioprinted, but cultured in 2D tissue culture treated plastic wells for 24 (left) and 72 h. (right) respectively. FL9 INT on the Y-axis corresponds to the 'fluorescent dye intensity' in both the figures.

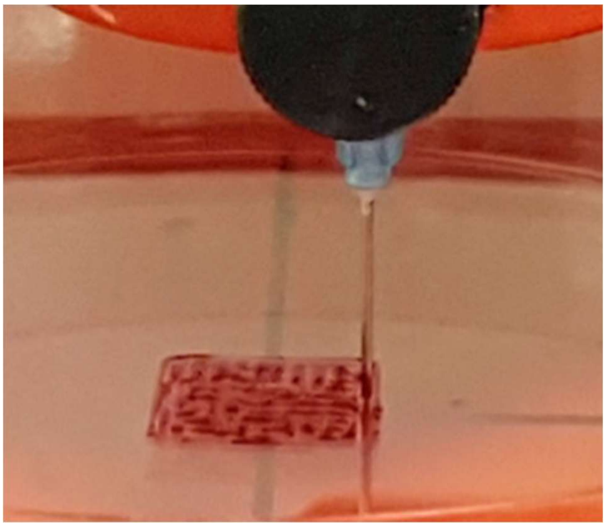

Supplementary Figure 3. Shown above is a lattice structure being printed, depicting horizontal and vertical lines which is believed to render macro-porosity to the structure.

Methods: For estimation of % apparent porosity, the software program Image J was used. For this, representative SEM images were acquired and analysed using Image J, as described. Images depicting cross sectional views of the samples were used. First the images were calibrated to convert a known distance in pixels to micron units. Next, the entire image area was traced out using the polygon selection tool and the total area estimated to determine 'total sample area of the cross section (sq.μm)'. The pores in images were identified and their area estimated separately to determine 'total area covered by pores (sq.μm)'. In addition, the average pore size was determined by finding the mean of all the pore lengths. The apparent porosity was determined by using the following formulae and reported as %, as done by other published studies [1–3]:

$$App.porosity = \frac{total\ area\ covered\ by\ pores\ (sq.\mu m)}{total\ sample\ area\ of\ the\ crosssection(sq.\mu m)} * 100$$

References:

1. Al Marzooqi, Faisal A., M. R. Bilad, Bilal Mansoor, and Hassan A. Arafat. "A comparative study of image analysis and porometry techniques for characterization of porous membranes." *Journal of materials science* 51, no. 4 (2016): 2017-2032.

2. Anil Kumar, Shweta, Shane C. Allen, Nishat Tasnim, Tahmina Akter, Shinhye Park, Alok Kumar, Munmun Chattopadhyay, Yoshihiro Ito, Laura J. Suggs, and Binata Joddar. "The applicability of furfuryl-gelatin as a novel bioink for tissue engineering applications." *Journal of Biomedical Materials Research Part B: Applied Biomaterials* (2018).

3. Tasnim, Nishat, Vikram Thakur, Munmun Chattopadhyay, and Binata Joddar. "The efficacy of Graphene-foams for culturing mesenchymal stem cells and differentiation into dopaminergic neurons." *Stem Cells International* 2018:1-12.
